# Hidden danger: silicosis in a dental technician—an overlooked occupational risk

**DOI:** 10.1093/omcr/omaf078

**Published:** 2025-06-27

**Authors:** Basma Beqqali, Ola Messaoud, Basma Dghoughi, Joud Boutaleb, Oumaima Mesbah, Omar El Aoufir, Laila Jroundi, Zaynab Iraqi Houssaini

**Affiliations:** Emergency radiology department, Ibn Sina Hospital, Mohammed V University, Rabat, Morocco; Emergency radiology department, Ibn Sina Hospital, Mohammed V University, Rabat, Morocco; Emergency radiology department, Ibn Sina Hospital, Mohammed V University, Rabat, Morocco; Emergency radiology department, Ibn Sina Hospital, Mohammed V University, Rabat, Morocco; Emergency radiology department, Ibn Sina Hospital, Mohammed V University, Rabat, Morocco; Emergency radiology department, Ibn Sina Hospital, Mohammed V University, Rabat, Morocco; Emergency radiology department, Ibn Sina Hospital, Mohammed V University, Rabat, Morocco; Emergency radiology department, Ibn Sina Hospital, Mohammed V University, Rabat, Morocco

**Keywords:** radiology, respiratory disorders

## Abstract

Silicosis is a chronic lung disease caused by prolonged inhalation of crystalline silica particles, frequently encountered in professionals exposed to silica dust. We report the case of a 54-year-old dental technician presenting with progressive dyspnea, where the diagnosis of silicosis was confirmed based on occupational history and characteristic radiological findings. A differential diagnosis with sarcoidosis was initially considered. This case highlights the importance of early recognition of occupational lung diseases and emphasizes the need for preventive measures to limit silica exposure.

## Introduction

Silicosis is a fibrotic lung disease that occurs due to chronic exposure to crystalline silica dust. It is classified into acute, accelerated, and chronic forms, with the latter being the most common. The pathophysiology involves inhaled silica particles inducing alveolar macrophage activation, inflammation, and progressive fibrosis. Despite advancements in workplace safety regulations, silicosis remains a significant occupational health concern.

**Figure 1:**
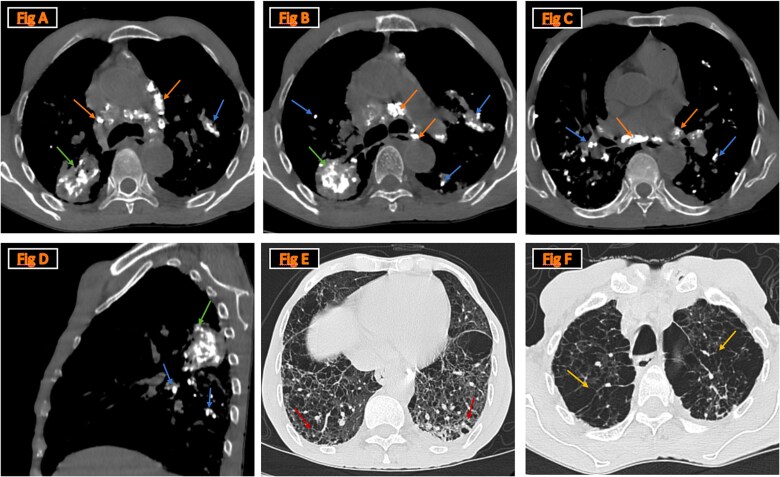
The CT scan images illustrate key radiologic features of silicosis: Mediastinal lymphadenopathy with eggshell calcifications, a hallmark of silicosis, is visible (orange arrows in figures A, B, and C). A fibrotic calcified mass in the right upper lobe is prominent (green arrows in figures A, B, and D). Calcified nodules and micronodules distributed randomly across the lung fields are highlighted (blue arrows in figures A, B, C and D). Diffuse fibrotic changes are visible, particularly in the lower lung fields with honeycombing, indicating fibrotic progression, is most pronounced at the lung bases (red arrows in fig. E). Upper lobe emphysematous changes, consistent with chronic silica exposure, are evident (yellow arrows in fig. F). These findings together confirm the diagnosis of silicosis and demonstrate its mixed presentation, including fibrosis, emphysema, and calcifications.

## Case presentation

A 54-year-old male dental technician, with a professional history spanning over 25 years, presented with progressive exertional dyspnea over several months. He denied productive cough, hemoptysis, or systemic symptoms such as fever or weight loss. There was no history of tuberculosis or autoimmune diseases.

On physical examination, decreased breath sounds were noted at the lung bases without crackles or wheezing. No signs of right heart failure or peripheral lymphadenopathy were observed.

The chest CT scan revealed as schown in Fig. 1, a fibrotic, calcified mass in the dorsal segment of the right upper lobe, alongside multiple calcified small pulmonary nodules with a perilymphatic distribution. These nodules were most pronounced in both lungs’ fissural regions, which are symmetrically distributed particularly at the bilateral Fowler zones. Mediastinal lymphadenopathy was also observed, demonstrating an ‘eggshell’ calcification pattern, a characteristic finding highly suggestive of silicosis [1].

Additionally, emphysema was noted, predominantly affecting the upper lobes. The scan revealed areas of honeycombing and fibrotic changes localized mainly to the lung bases, suggesting advanced structural damage to the parenchyma. These findings, in conjunction with the patient’s exposure history and clinical presentation, provided critical diagnostic insights.

**A Pulmonary function tests (PFTs):** Showed a mild restrictive pattern with a reduced diffusion capacity for carbon monoxide (DLCO).

**Arterial blood gases:** Normal oxygenation at rest with mild desaturation on exertion.

**Serologic markers:** Autoantibody screening was negative.

Tuberculin skin test and QuantiFERON-TB Gold: Negative results.

**Mediastinoscopy with lymph node biopsy:** Revealed fibrosis without non-caseating granulomas, excluding sarcoidosis. **This important diagnostic step allowed the authors to rule out sarcoidosis as a differential diagnosis. Given the clinical and radiological similarities between sarcoidosis and silicosis, the use of histological confirmation provided robust support to the diagnosis and reinforced the specificity of the findings attributed to silicosis.**

## Discussion

The diagnosis of chronic silicosis was confirmed based on the characteristic radiological findings and the patient’s prolonged occupational exposure. Silicosis is a well-documented pneumoconiosis in workers exposed to silica dust, particularly in mining [2], quarrying, and the ceramics industry. However, it is **less recognized in dental technicians**, despite their exposure to silica dust during prosthesis polishing and grinding. This lack of awareness can lead to delayed diagnosis, emphasizing the need for greater awareness and **preventive measures** tailored to this profession.

Silicosis presents a radiological and clinical pattern that may be confused with other interstitial or granulomatous lung diseases. In this case, the **main diagnostic challenge** was differentiating between silicosis and **sarcoidosis**, given the presence of mediastinal lymphadenopathy and pulmonary nodules. **Although the clinical presentation raised suspicion of sarcoidosis, the mediastinoscopy with lymph node biopsy confirmed the absence of non-caseating granulomas, thereby excluding sarcoidosis. This clarification is crucial in establishing the correct diagnosis and directing the appropriate management strategy. Cases of concomitant silicosis and sarcoidosis (‘silicosarcoidosis**’) have been reported, highlighting the need for histopathological confirmation in ambiguous cases.

Beyond sarcoidosis, the differential diagnosis includes pulmonary tuberculosis [3], pneumoconioses caused by other mineral particles, and chronic necrotizing pulmonary aspergillosis. **Tuberculosis is particularly common in patients with silicosis** due to impaired pulmonary immunity [4]; however, in this case, microbiological tests were negative. Pneumoconioses caused by other particles, such as **coal worker’s pneumoconiosis or berylliosis** [1], share clinical and radiological similarities, but the patient’s occupational history and the specific nature of his exposure helped guide the diagnosis.

The presence of emphysema, more pronounced in the upper lobes, in our patient suggests a mixed pattern of lung injury possibly due to prolonged inhalation of silica dust [5]. Honeycombing at the lung bases indicates fibrotic progression, resembling chronic interstitial lung disease. Silicosis remains a significant occupational health concern, particularly in low-income countries, where tuberculosis and silica-related lung diseases remain prevalent. The disease is irreversible and associated with an increased risk of progressive pulmonary fibrosis, chronic respiratory failure, and malignancy.

There is no curative treatment; management is based on avoiding further silica exposure, symptomatic relief, and regular follow-up to monitor disease progression. Treatment is symptomatic, including the use of bronchodilators and oxygen therapy in cases of chronic respiratory failure [6]. Advanced cases may require pulmonary rehabilitation or, in severe cases, lung transplantation. Preventive measures, such as workplace ventilation, use of personal protective equipment (PPE), and periodic health surveillance, are essential to reduce occupational exposure. Raising awareness among healthcare professionals about occupational lung diseases can aid in early diagnosis and intervention.

## Conclusion

Pulmonary silicosis remains a prevalent occupational lung disease, particularly in workers exposed to silica dust, such as dental technicians. This case emphasizes the importance of considering occupational exposure in respiratory disorders and highlights the role of **radiologic and histologic findings** in differentiating silicosis from other granulomatous lung diseases like sarcoidosis [7]. Early recognition and preventive strategies are essential to reducing disease burden and improving patient outcomes.
